# Supplementary material for: Toward a better definition of hematopoietic progenitors suitable for B cell differentiation
Source: PLoS One. 2020 Dec 15;15(12):e0243769. doi: 10.1371/journal.pone.0243769 (PMC7737978; doi:10.1371/journal.pone.0243769)
Supplement: Attachment — Submitted filename: Response to Reviewers.docx [file pone.0243769.s004.docx]

18^th^ October 2020

Dear Editor,

We are hereby submitting our revised version of the manuscript entitled “*Toward a better definition of hematopoietic progenitors suitable for B cell differentiation*”.

We addressed all the comments of the referees and we have amended the manuscript accordingly. Because of the actual sanitary restriction, we had to stop our investigation and we were not able to performed new B cell differentiation to further characterize iPSC. However, all the points have been addressed and all the new analysis required have been done.

We believe that these changes have significantly improved the article. A point-by-point reply is provided and the modifications in the manuscript are indicated in the track-changes version.

We thank editor and reviewers for giving us the chance to resubmit our work and hope that this manuscript is now acceptable for publication.

We look forward to hearing from you.

Yours sincerely,

Dr. S. Brouard & L. David

**Reviewer #1:** The authors compared CD34+ progenitors derived from cord blood and human ESC. They identified CD31(int)CD45(int) as efficient hematopoietic progenitors suitable for B cell differentiation. Interestingly, human PSCs are less efficient to generate B cells than cord blood stem cells.

Major concerns:

1. In many experiments or conditions, there is only 1 independent experiment and there are no solid statistical comparison to reach any conclusion.

For more clarity, we chose to only show the experiments performed with one cytokine concentration of 10ng/mL in Figure 2B and 2C. Nevertheless, this experiment with the condition D was reproduced several times with cytokines used at 25ng/mL (Figure 3A, n=3). As mentioned by reviewer 2, we performed statistical tests (Significance of difference between nonparametric variables were assessed with a Mann-Whitney test and the differences were considered statistically significant at p < 0.05) and confirmed statistical difference between the conditions and the CTRL in the figure 2, 3 and S1. This is now mentioned in the manuscript page 10 in the material and methods section. The results section (page 12-13) and the figures 2, 3 and S1 have been up-dated in consequence.

2. In the first 10 days derivation of CD34+ cell from hPSCs, although the authors tried to play with different concentrations or combinations of cytokines to get the highest efficiency, the transition from hPSCs into specific mesoderm progenitors and then differentiate into CD34+ progenitors are overlooked. Certain mesoderm progenitors (i.e., anterior or posterior) favor the hematopoietic differentiation.

Reviewer 1 is right. We focused on previously validated differentiation culture conditions on OP9, with CD34+ / hematopoietic engagement as our main readout [1,2]. Reviewer 1 raises an interesting perspective to further improve our protocol. We have added this perspective in the discussion of our manuscript (page 23).

3. The authors discussed a lot of efficienty difference and the functional characterization of the differentiated B cells except for B cell markers is missing

Given the low efficiency of B cell differentiation, we focused our study on the characterization of intermediate stages. We think this data can be used as readouts to tweak the differentiation protocol. Unfortunately, the few amount of B cells generated did not permit any sorting and functional characterisation, we first need to optimise the protocol and reach a sufficient number of B cells. This point has been mentioned in the new version of the manuscript page 22 in the discussion section.

**Reviewer #2:** In this manuscript, Florian Dubois et al. reported that they were interested in the relationship between B cells and hPSCs-derived hematopoietic stem cells. They differentiated the hPSCs into hematopoietic stem cells through the OP9stromal cell, and the B cells were generated on MS-5 stromal cells. They investigated the influence of various cytokine conditions on the hematopoietic stem cell generation, and they highlight CD31intCD45int phenotype as a possible marker of hematopoietic progenitors suitable for B cell differentiation. This manuscript should be revised as below.

Major modification:

(1) At the introduction part, they authors should make more illustration about the relationship between B cells and hematopoietic stem cells, especially the published protocols for B cell differentiation.

We thank the reviewer for his/her suggestion and have expended our introduction accordingly as followed: “B cell differentiation occurs in the bone marrow from HSC through different steps and a complex transcriptional process yet well characterized involving different cytokines [3,4]. Previous rapport highlighted the efficiency of in vitro B cell differentiation from cord blood CD34^+^ progenitors, as a source of HSC, on a monolayer of MS-5 or S17 stromal cells [5-7]. However, committing hPSC toward B cells remains a challenging process mainly due to the difficulty to generate proper hematopoietic stem cells from hPSC.”. This part has been added to the manuscript in the introduction section page 4.

(2) At the experiment section, the OP9 cells were cultured in αMEM medium instead of aMEM medium.

We double-checked and we confirmed that OP9 cells were cultured in the right αMEM medium (without nucleoside) supplemented with fetal bovine serum to a final concentration of 20% as recommended by ATCC; Vodyanick et al., 2005; Carpenter et al., 2011; and French et al., 2015. We have replaced all “aMEM” occurrences with “αMEM”.

(3) The authors should perform further characterization on the iPSC-derived hematopoietic stem/progenitor cells, the characterization is not enough.

We agree with that. Unfortunately, due to sanitary restriction, we had to stop our investigation and we were not able to performed new B cell differentiation to further characterize iPSC-derived hematopoietic stem/progenitor cells. We hope that reviewer will be sensible to this point.

(4) As a biological experiment data, the authors should make additional statistical analysis, for example, Fig 2-7.

To answer reviewer 2, we have performed statistical analysis for all experiments with n > 3. The material and method section (page 10), the results section (page 12-13) and the figures 2, 3 and S1 have been up-dated in consequence.

(5) In order to make this manuscript better, the authors should improve the layout quality of the Figures, especially Fig 5.

Accordingly, the layout quality of all figures has been improved as required.

(6) The authors should make a scheme illustration to present their key ideas or results.

As required by reviewer 2 and to clarify our results, we added a scheme illustration of the B cell differentiation key steps in figure 1A and figure S3.
